# Dimethyl 3-phenyl­penta­nedioate

**DOI:** 10.1107/S1600536810041954

**Published:** 2010-11-10

**Authors:** Peng Zhang, Feng Fu, Ni Wang

**Affiliations:** a Department of Chemistry and Chemical Engineering, Shaanxi Key Laboratory of Chemical Reaction Engineering, Yan’an University, Shaanxi 716000, People’s Republic of China

## Abstract

In the title compound, C_13_H_16_O_4_, the terminal carboxyl­ate groups are twisted to each other at a dihedral angle of 23.80 (9)°. Weak inter­molecular C—H⋯O hydrogen bonds link the mol­ecules into supra­molecular chains along the *a* axis.

## Related literature

For approximately extended structures of carbon skeleton in penta­nedioate compounds, see: Fun & Chantrapromma (2009 ▶); Karadayı (2008 ▶); Yang *et al.* (2008 ▶).
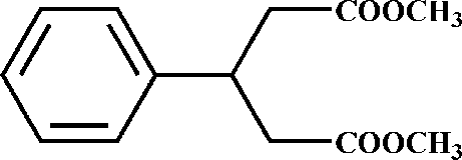


## Experimental

### 

#### Crystal data

C_13_H_16_O_4_
*M* *_r_* = 236.26
Triclinic, 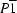

*a* = 5.7944 (2) Å
*b* = 8.7668 (3) Å
*c* = 12.7591 (4) Å
α = 92.609 (2)°
β = 101.979 (2)°
γ = 96.140 (2)°
*V* = 628.86 (4) Å^3^
*Z* = 2
Mo *K*α radiation
μ = 0.09 mm^−1^
*T* = 296 K
0.32 × 0.26 × 0.13 mm

#### Data collection

Bruker SMART 1000 CCD diffractometer
8883 measured reflections
2207 independent reflections
1839 reflections with *I* > 2σ(*I*)
*R*_int_ = 0.020

#### Refinement

*R*[*F* ^2^ > 2σ(*F* ^2^)] = 0.038
*wR*(*F* ^2^) = 0.116
*S* = 1.07
2207 reflections
156 parameters
H-atom parameters constrained
Δρ_max_ = 0.15 e Å^−3^
Δρ_min_ = −0.14 e Å^−3^

### 

Data collection: *SMART* (Bruker, 1997 ▶); cell refinement: *SAINT* (Bruker, 1997 ▶); data reduction: *SAINT*; program(s) used to solve structure: *SHELXTL* (Sheldrick, 2008 ▶); program(s) used to refine structure: *SHELXTL*; molecular graphics: *SHELXTL*; software used to prepare material for publication: *SHELXTL*.

## Supplementary Material

Crystal structure: contains datablocks I, global. DOI: 10.1107/S1600536810041954/xu5052sup1.cif

Structure factors: contains datablocks I. DOI: 10.1107/S1600536810041954/xu5052Isup2.hkl

Additional supplementary materials:  crystallographic information; 3D view; checkCIF report

## Figures and Tables

**Table 1:** Hydrogen-bond geometry (Å, °)

*D*—H⋯*A*	*D*—H	H⋯*A*	*D*⋯*A*	*D*—H⋯*A*
C8—H8*B*⋯O4^i^	0.97	2.53	3.3987 (19)	149

Symmetry code: (i) 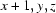.

**Fig. 1.**
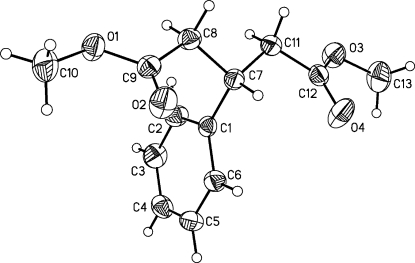
The molecular structure of the title compound, showing the atomic numbering scheme. The displacement ellipsoids are drawn at the 50% probability level.

**Fig. 2.**
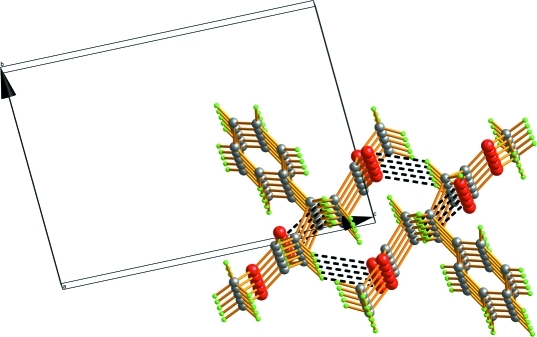
One-dimensional double chain connected by hydrogen bonds in the title complex.

## supplementary crystallographic information

### Comment

We attempted to synthesize a Cd^II^ complex with the mixed ligands using hydrothermal conditions. However we were not successful, and the title ester was unexpectedly obtained. Its structure is reported here.

The molecular structure is shown in Fig 1. The phenyl ring is almost perpendicular to the plane of C7, C8 and C11 atoms with a dihedral angle of 88.945 (6)°. The carbon skeleton of the pentanedioate displays an approximately extended structure, similar to those found in the crystal structures of related compounds (Fun & Chantrapromma, 2009; Karadayı, 2008; Yang *et al.* 2008). The terminal carboxylate groups are twisted to each other with a dihedal angle of 23.80 (9)°.

In the crystal structure, molecules are linked by C–H···O hydrogen bonds into a one-dimensional chain along the *a* axis (Table 1).

### Experimental

All chemicals were of reagent grade quality obtained from commercial sources and used without further purification. An H_2_O/CH_3_OH (1:1) solution (8.0 ml) containing mixture of Cd(NO_3_)_2_.6H_2_O (0.1 mmol, 0.0308 mg), 3-phenylglutaric acid (0.1 mmol, 0.0208 g), 1,3-di(4-pyridyl)propane (0.05 mmol, 0.0099 g) and NaOH (0.1 mmol) was sealed in a 23 ml Teflon-lined autoclave, heated at 413 K for 3 days and then cooled to room temperature at 5 K min^-1^. The colorless crystals were obtained.

### Refinement

All of the H atoms were positioned geometrically [C—H = 0.93–0.96 Å] and refined using a riding model with U_iso_(H)= 1.2 or 1.5U_eq_(C).

### Crystal data

**Table:**

C_13_H_16_O_4_	*Z* = 2
*M**_r_* = 236.26	*F*(000) = 252
Triclinic, *P*1	*D*_x_ = 1.248 Mg m^−^^3^
Hall symbol: -P 1	Mo *K*α radiation, λ = 0.71073 Å
*a* = 5.7944 (2) Å	Cell parameters from 2207 reflections
*b* = 8.7668 (3) Å	θ = 1.6–25.0°
*c* = 12.7591 (4) Å	µ = 0.09 mm^−^^1^
α = 92.609 (2)°	*T* = 296 K
β = 101.979 (2)°	Prism, colorless
γ = 96.140 (2)°	0.32 × 0.26 × 0.13 mm
*V* = 628.86 (4) Å^3^	

### Data collection

**Table:**

Bruker SMART 1000 CCD diffractometer	1839 reflections with *I* > 2σ(*I*)
Radiation source: fine-focus sealed tube	*R*_int_ = 0.020
graphite	θ_max_ = 25.0°, θ_min_ = 1.6°
φ and ω scans	*h* = −6→6
8883 measured reflections	*k* = −9→10
2207 independent reflections	*l* = −15→14

### Refinement

**Table:**

Refinement on *F*^2^	Primary atom site location: structure-invariant direct methods
Least-squares matrix: full	Secondary atom site location: difference Fourier map
*R*[*F*^2^ > 2σ(*F*^2^)] = 0.038	Hydrogen site location: inferred from neighbouring sites
*wR*(*F*^2^) = 0.116	H-atom parameters constrained
*S* = 1.07	*w* = 1/[σ^2^(*F*_o_^2^) + (0.0636*P*)^2^ + 0.0827*P*] where *P* = (*F*_o_^2^ + 2*F*_c_^2^)/3
2207 reflections	(Δ/σ)_max_ < 0.001
156 parameters	Δρ_max_ = 0.15 e Å^−^^3^
0 restraints	Δρ_min_ = −0.14 e Å^−^^3^

### Special details

**Table:**

Geometry. All e.s.d.'s (except the e.s.d. in the dihedral angle between two l.s. planes) are estimated using the full covariance matrix. The cell e.s.d.'s are taken into account individually in the estimation of e.s.d.'s in distances, angles and torsion angles; correlations between e.s.d.'s in cell parameters are only used when they are defined by crystal symmetry. An approximate (isotropic) treatment of cell e.s.d.'s is used for estimating e.s.d.'s involving l.s. planes.
Refinement. Refinement of *F*^2^ against ALL reflections. The weighted *R*-factor *wR* and goodness of fit *S* are based on *F*^2^, conventional *R*-factors *R* are based on *F*, with *F* set to zero for negative *F*^2^. The threshold expression of *F*^2^ > σ(*F*^2^) is used only for calculating *R*-factors(gt) *etc*. and is not relevant to the choice of reflections for refinement. *R*-factors based on *F*^2^ are statistically about twice as large as those based on *F*, and *R*- factors based on ALL data will be even larger.

### Fractional atomic coordinates and isotropic or equivalent isotropic displacement parameters (Å^2^)

**Table:**

	*x*	*y*	*z*	*U*_iso_*/*U*_eq_	
C1	0.4786 (2)	0.22914 (15)	0.76660 (10)	0.0402 (3)	
C2	0.6254 (3)	0.26207 (18)	0.69556 (13)	0.0551 (4)	
H2	0.7299	0.1932	0.6833	0.066*	
C3	0.6182 (3)	0.3963 (2)	0.64272 (13)	0.0635 (5)	
H3	0.7184	0.4169	0.5955	0.076*	
C4	0.4658 (3)	0.49892 (18)	0.65906 (13)	0.0579 (4)	
H4	0.4610	0.5886	0.6229	0.069*	
C5	0.3203 (3)	0.46854 (17)	0.72911 (13)	0.0578 (4)	
H5	0.2166	0.5382	0.7410	0.069*	
C6	0.3263 (3)	0.33451 (16)	0.78254 (12)	0.0496 (4)	
H6	0.2261	0.3152	0.8299	0.060*	
C7	0.4854 (2)	0.08246 (15)	0.82520 (11)	0.0427 (3)	
H7	0.3652	0.0799	0.8692	0.051*	
C8	0.7273 (3)	0.07618 (17)	0.89975 (11)	0.0488 (4)	
H8A	0.7261	−0.0220	0.9319	0.059*	
H8B	0.8482	0.0813	0.8572	0.059*	
C9	0.7936 (3)	0.20195 (16)	0.98715 (11)	0.0463 (3)	
C10	1.1203 (4)	0.3609 (2)	1.09571 (16)	0.0793 (6)	
H10A	1.0243	0.4439	1.0931	0.119*	
H10B	1.2801	0.4007	1.0937	0.119*	
H10C	1.1199	0.3104	1.1609	0.119*	
C11	0.4293 (2)	−0.06163 (16)	0.74809 (12)	0.0487 (4)	
H11A	0.5381	−0.0554	0.6996	0.058*	
H11B	0.4573	−0.1507	0.7892	0.058*	
C12	0.1809 (3)	−0.08498 (16)	0.68321 (12)	0.0474 (3)	
C13	−0.0740 (4)	−0.2120 (3)	0.52859 (17)	0.0895 (6)	
H13A	−0.1606	−0.2865	0.5633	0.134*	
H13B	−0.0641	−0.2549	0.4593	0.134*	
H13C	−0.1546	−0.1218	0.5202	0.134*	
O1	1.02509 (18)	0.25234 (13)	1.00486 (9)	0.0591 (3)	
O2	0.6623 (2)	0.25035 (15)	1.03815 (10)	0.0713 (4)	
O3	0.1625 (2)	−0.17144 (14)	0.59333 (9)	0.0671 (3)	
O4	0.0144 (2)	−0.03577 (17)	0.70777 (11)	0.0850 (5)	

### Atomic displacement parameters (Å^2^)

**Table:**

	*U*^11^	*U*^22^	*U*^33^	*U*^12^	*U*^13^	*U*^23^
C1	0.0367 (7)	0.0427 (7)	0.0383 (7)	0.0014 (6)	0.0042 (5)	−0.0019 (6)
C2	0.0524 (9)	0.0602 (9)	0.0589 (9)	0.0142 (7)	0.0209 (7)	0.0101 (7)
C3	0.0660 (10)	0.0697 (11)	0.0601 (10)	0.0054 (9)	0.0240 (8)	0.0175 (8)
C4	0.0665 (10)	0.0476 (8)	0.0548 (9)	0.0017 (7)	0.0033 (8)	0.0099 (7)
C5	0.0613 (10)	0.0451 (8)	0.0673 (10)	0.0143 (7)	0.0109 (8)	0.0005 (7)
C6	0.0500 (8)	0.0464 (8)	0.0549 (9)	0.0067 (6)	0.0167 (7)	0.0011 (7)
C7	0.0403 (7)	0.0432 (7)	0.0441 (7)	0.0044 (6)	0.0085 (6)	0.0010 (6)
C8	0.0484 (8)	0.0486 (8)	0.0477 (8)	0.0108 (6)	0.0041 (6)	0.0026 (6)
C9	0.0467 (8)	0.0503 (8)	0.0414 (7)	0.0086 (6)	0.0061 (6)	0.0080 (6)
C10	0.0702 (12)	0.0790 (12)	0.0741 (12)	−0.0042 (10)	−0.0060 (10)	−0.0174 (10)
C11	0.0474 (8)	0.0435 (8)	0.0542 (8)	0.0066 (6)	0.0083 (7)	−0.0007 (6)
C12	0.0487 (8)	0.0417 (7)	0.0511 (8)	0.0011 (6)	0.0120 (7)	−0.0007 (6)
C13	0.0772 (13)	0.0980 (15)	0.0754 (13)	−0.0134 (11)	−0.0073 (10)	−0.0194 (11)
O1	0.0474 (6)	0.0657 (7)	0.0592 (7)	0.0014 (5)	0.0048 (5)	−0.0079 (5)
O2	0.0604 (7)	0.0865 (9)	0.0668 (7)	0.0044 (6)	0.0206 (6)	−0.0181 (6)
O3	0.0603 (7)	0.0713 (7)	0.0625 (7)	0.0036 (6)	0.0047 (6)	−0.0208 (6)
O4	0.0469 (7)	0.1122 (11)	0.0901 (9)	0.0096 (7)	0.0109 (6)	−0.0360 (8)

### Geometric parameters (Å, °)

**Table:**

C1—C6	1.3803 (18)	C8—H8B	0.9700
C1—C2	1.3855 (19)	C9—O2	1.1962 (17)
C1—C7	1.5166 (18)	C9—O1	1.3357 (18)
C2—C3	1.383 (2)	C10—O1	1.441 (2)
C2—H2	0.9300	C10—H10A	0.9600
C3—C4	1.364 (2)	C10—H10B	0.9600
C3—H3	0.9300	C10—H10C	0.9600
C4—C5	1.366 (2)	C11—C12	1.492 (2)
C4—H4	0.9300	C11—H11A	0.9700
C5—C6	1.385 (2)	C11—H11B	0.9700
C5—H5	0.9300	C12—O4	1.1908 (18)
C6—H6	0.9300	C12—O3	1.3244 (18)
C7—C11	1.5283 (19)	C13—O3	1.444 (2)
C7—C8	1.5299 (19)	C13—H13A	0.9600
C7—H7	0.9800	C13—H13B	0.9600
C8—C9	1.492 (2)	C13—H13C	0.9600
C8—H8A	0.9700		
			
C6—C1—C2	117.79 (13)	C7—C8—H8B	108.7
C6—C1—C7	121.06 (12)	H8A—C8—H8B	107.6
C2—C1—C7	121.15 (12)	O2—C9—O1	123.14 (14)
C3—C2—C1	120.70 (14)	O2—C9—C8	125.79 (14)
C3—C2—H2	119.7	O1—C9—C8	111.03 (12)
C1—C2—H2	119.7	O1—C10—H10A	109.5
C4—C3—C2	120.75 (15)	O1—C10—H10B	109.5
C4—C3—H3	119.6	H10A—C10—H10B	109.5
C2—C3—H3	119.6	O1—C10—H10C	109.5
C3—C4—C5	119.34 (14)	H10A—C10—H10C	109.5
C3—C4—H4	120.3	H10B—C10—H10C	109.5
C5—C4—H4	120.3	C12—C11—C7	114.15 (11)
C4—C5—C6	120.37 (14)	C12—C11—H11A	108.7
C4—C5—H5	119.8	C7—C11—H11A	108.7
C6—C5—H5	119.8	C12—C11—H11B	108.7
C1—C6—C5	121.05 (14)	C7—C11—H11B	108.7
C1—C6—H6	119.5	H11A—C11—H11B	107.6
C5—C6—H6	119.5	O4—C12—O3	122.43 (15)
C1—C7—C11	112.33 (11)	O4—C12—C11	125.58 (14)
C1—C7—C8	111.83 (11)	O3—C12—C11	111.98 (12)
C11—C7—C8	108.24 (11)	O3—C13—H13A	109.5
C1—C7—H7	108.1	O3—C13—H13B	109.5
C11—C7—H7	108.1	H13A—C13—H13B	109.5
C8—C7—H7	108.1	O3—C13—H13C	109.5
C9—C8—C7	114.04 (11)	H13A—C13—H13C	109.5
C9—C8—H8A	108.7	H13B—C13—H13C	109.5
C7—C8—H8A	108.7	C9—O1—C10	117.15 (13)
C9—C8—H8B	108.7	C12—O3—C13	116.62 (14)
			
C6—C1—C2—C3	0.0 (2)	C1—C7—C8—C9	61.88 (15)
C7—C1—C2—C3	−179.85 (14)	C11—C7—C8—C9	−173.87 (12)
C1—C2—C3—C4	−0.3 (3)	C7—C8—C9—O2	40.5 (2)
C2—C3—C4—C5	0.5 (3)	C7—C8—C9—O1	−141.58 (13)
C3—C4—C5—C6	−0.4 (3)	C1—C7—C11—C12	−66.14 (15)
C2—C1—C6—C5	0.1 (2)	C8—C7—C11—C12	169.91 (12)
C7—C1—C6—C5	179.92 (14)	C7—C11—C12—O4	−24.7 (2)
C4—C5—C6—C1	0.1 (2)	C7—C11—C12—O3	156.52 (12)
C6—C1—C7—C11	121.25 (14)	O2—C9—O1—C10	5.2 (2)
C2—C1—C7—C11	−58.94 (17)	C8—C9—O1—C10	−172.73 (14)
C6—C1—C7—C8	−116.81 (14)	O4—C12—O3—C13	−3.8 (2)
C2—C1—C7—C8	62.99 (17)	C11—C12—O3—C13	175.03 (15)

### Hydrogen-bond geometry (Å, °)

**Table:**

*D*—H···*A*	*D*—H	H···*A*	*D*···*A*	*D*—H···*A*
C8—H8B···O4^i^	0.97	2.53	3.3987 (19)	149

Symmetry codes: (i) *x*+1, *y*, *z*.
